# Isolated Myositis Unveiling Systemic Lupus Erythematosus: A Rare Initial Presentation

**DOI:** 10.7759/cureus.88269

**Published:** 2025-07-18

**Authors:** Amit Kumar, Sandeep Garg, Praveen Bharti, Aarti Sah, Bhvika Zutshi

**Affiliations:** 1 General Medicine, Maulana Azad Medical College, New Delhi, IND; 2 Medicine, Lok Nayak Hospital, Maulana Azad Medical College, New Delhi, IND; 3 Medicine, Maulana Azad Medical College, New Delhi, IND

**Keywords:** autoimmune disease, case report, inflammatory myopathy, muscle biopsy, myositis, systemic lupus erythematosus

## Abstract

Systemic lupus erythematosus (SLE) is a heterogeneous autoimmune disease with a broad range of clinical manifestations. While musculoskeletal involvement is common in SLE, myositis as the initial and isolated presentation is rare and can lead to diagnostic delays.

We report the case of a 19-year-old female who presented with progressive, symmetrical proximal muscle weakness over several weeks, without accompanying joint, cutaneous, or systemic symptoms. Laboratory workup revealed elevated serum creatine kinase and positive antinuclear antibodies. Electromyography findings were suggestive of inflammatory myopathy, and muscle biopsy confirmed autoimmune myositis. Further autoimmune testing confirmed the presence of anti-dsDNA and anti-Sm antibodies. Based on the 2019 EULAR/ACR classification criteria, a diagnosis of SLE with predominant myositis was established. The patient showed significant clinical improvement following the initiation of corticosteroids and hydroxychloroquine.

This case underscores the importance of considering SLE in the differential diagnosis of inflammatory myopathies, especially in young females presenting with isolated proximal muscle weakness. Early recognition and treatment of lupus-associated myositis can prevent disease progression and improve outcomes.

## Introduction

Systemic lupus erythematosus (SLE) is a multifaceted, chronic autoimmune disorder characterized by a wide range of clinical presentations, predominantly impacting young females. This condition is marked by the generation of pathogenic autoantibodies targeting nuclear, cytoplasmic, and cell surface antigens, leading to immune complex formation and multi-organ inflammation [1]. The conventional manifestations encompass constitutional symptoms, skin lesions, arthritis, nephritis, and hematological abnormalities; nevertheless, the illness may exhibit unusual aspects in certain people.

Musculoskeletal symptoms are prevalent clinical characteristics of SLE, affecting roughly 70%-90% of patients throughout the illness progression [2]. The standard manifestation includes non-erosive arthritis or arthralgia. Myositis, characterized by inflammation of skeletal muscle, is an uncommon presentation in SLE, occurring in around 4%-16% of patients, and is even less frequently the initial presenting symptom [3,4].

In SLE, myositis can clinically resemble primary idiopathic inflammatory myopathies, including polymyositis (PM) and dermatomyositis (DM). These disorders exhibit clinical characteristics including symmetrical proximal muscular weakness, increased muscle enzymes (creatine kinase (CK) and aldolase), aberrant electromyography (EMG) results, and inflammatory infiltrates shown in muscle biopsies [5]. In contrast to intrinsic myopathies, SLE-associated myositis may exhibit milder symptoms, have a favorable response to corticosteroids, and is frequently accompanied by other systemic manifestations and positive serological findings for lupus-specific autoantibodies.

Identifying inflammatory myopathy as the initial and exclusive presenting symptom of SLE is essential for prompt diagnosis and treatment. Autoantibody testing, encompassing antinuclear antibodies (ANA), anti-double-stranded DNA (anti-dsDNA), and extractable nuclear antigen antibodies (e.g., anti-Sm), is essential for diagnosing the condition [6]. A muscle biopsy differentiates lupus myositis from other etiologies and verifies the inflammatory characteristics of the condition.

This case report details a unique and diagnostically complex case of SLE, in which the patient first had isolated inflammatory myopathy. This example emphasizes the necessity for a comprehensive autoimmune evaluation in individuals with unexplained proximal muscle weakness and illustrates the intersection between lupus and inflammatory myopathies.

## Case presentation

A 19-year-old female presented to the medicine department with chief complaints of weakness and pain in all four limbs, which was insidious in onset, gradually progressive, and the weakness was more pronounced in proximal muscles. The patient experienced difficulty with exercise, as well as weakness when climbing stairs or standing up from a chair. There was no history of fever, muscle cramps, rash on the body or around the eyes, photosensitivity, joint pain, weight loss, gastrointestinal symptoms, or similar complaints in family members. The patient also had no prior history of any illness. There were no other symptoms, including chronic dry eyes, difficulty swallowing, or exertional dyspnea. The patient reported pain and weakness predominantly affecting the muscles of the upper and lower extremities. On examination, higher mental functions were intact. There was power of 3/5 in all limbs without any atrophy on the proximal muscles and 5/5 in the distal muscles. Cranial nerve test results were normal, and there were no pathologic reflexes or lower motor neuron signs. There was no gait abnormality and no signs of cerebellar involvement. There was no rash, any skin lesions or periorbital lesions, and no evidence of Raynaud’s phenomenon on the skin of the fingers.

Laboratory tests indicated hemoglobin at 8.8 gm/dL, total leucocyte count at 6000/dL, platelet count at 2.6 lac, serum ferritin at 160 ng/dL, serum iron at 45 mcg/dL (normal range: 35-120 mcg/dL), total iron binding capacity (TIBC) at 274 (normal range: 162-261 mcg/dL), and transferrin saturation (TS%) below 20%. The peripheral smear indicates normocytic normochromic anemia, with elevated levels of CK (305 IU/L, normal range: 145) and lactate dehydrogenase (LDH 201 U/L, normal range: 247). The elevation of inflammatory markers was observed, with an erythrocyte sediment rate (ESR) of 128 mm/1st hour (normal range: 20 mm/1st hour) and C-reactive protein (CRP) of 72 mg/L (normal range: 5 mg/L). ANA were negative by indirect immunofluorescence (IF); however, SS-A/Ro60, SS-A/Ro52, and SS-B/La antibodies tested positive. Other connective tissue disease-related markers, including anti-RNP, anti-dsDNA, and anti-Scl-70, were negative. The myositis panel results indicated a 1+ positivity for Ku autoantibody and a 3+ positivity for RO-52 autoantibody. The results indicate the presence of non-specific myositis. The rheumatoid factor was mildly elevated at 16.7 IU/mL (normal range: 0-14), while the serum levels of complement factors C3 and C4 were within normal limits. A summary of the key laboratory findings is provided in Table 1.

**Table 1:** Key laboratory findings of the patient

Name of test	Measured values	Reference values
Hemoglobin	8.8 gm/dL	12-16 gm/dL
Total leucocyte counts	6000	3000-11000
Platelet count	2.6 lac	1.5-4.5 lac
Peripheral smear	Normocytic normochromic anemia	
T. Bilirubin	0.8 mg/dL	0.5-1.5 mg/dL
Aspartate aminotransferase (AST)	27 U/L	<35 U/L
Alanine aminotransferase (ALT)	25 U/L	<35 U/L
Alkaline phosphatase (ALP)	310 U/L	30-120 U/L
Total protein	7.4 g/dL	6.0-8.3 g/dL
Serum albumin	4.4 g/dL	3.5-5.5 g/dL
Blood urea	26 mg/dL	20-40 mg/dL
Creatinine	0.7 mg/dL	0.7-1.2 mg/dL
Creatine kinase	305 IU/L	<145 IU/dL
Lactate dehydrogenase (LDH)	201 U/L	120-246 U/L
Uric acid (UA)	4.3 mg/dL	3.5-8.5 mg/dL
Serum calcium	9.3 mg/dL	8-11 mg/dL
Serum phosphorus	3.1 mg/dL	2.5-4.5 MG/dL
Serum parathyroid hormone (PTH)	31 pg/mL	10-65 pg/mL
Serum ferritin	160 ng/dL	10-150 ng/dL
Serum iron	45 mcg/dL	35-120 mcg/dL
Total iron binding capacity (TIBC)	274	162-261 mcg/dL
Erythrocyte sediment rate (ESR)	128 mm/1^st^ hour	20 mm/1^st^ hour
C-reactive protein (CRP)	72 mg/L	<5 mg/L
Rheumatoid factor	16.7 IU/mL	<14 IU/mL
Serum complement factor (C3/C4)	Normal	
Antinuclear antibodies (ANA)	ANA were negative by indirect immunofluorescence (IF), but SS-A/Ro60 and SS-B/La were 4+, and other CTD-related markers, including anti-RNP, anti-dsDNA, and anti-Scl-70, were not found.	
Myositis panel	Ku autoantibody was 1+ positive, and RO-52 autoantibody was 3+ positive. These results are suggestive of non-specific myositis.	

**Figure 1:**
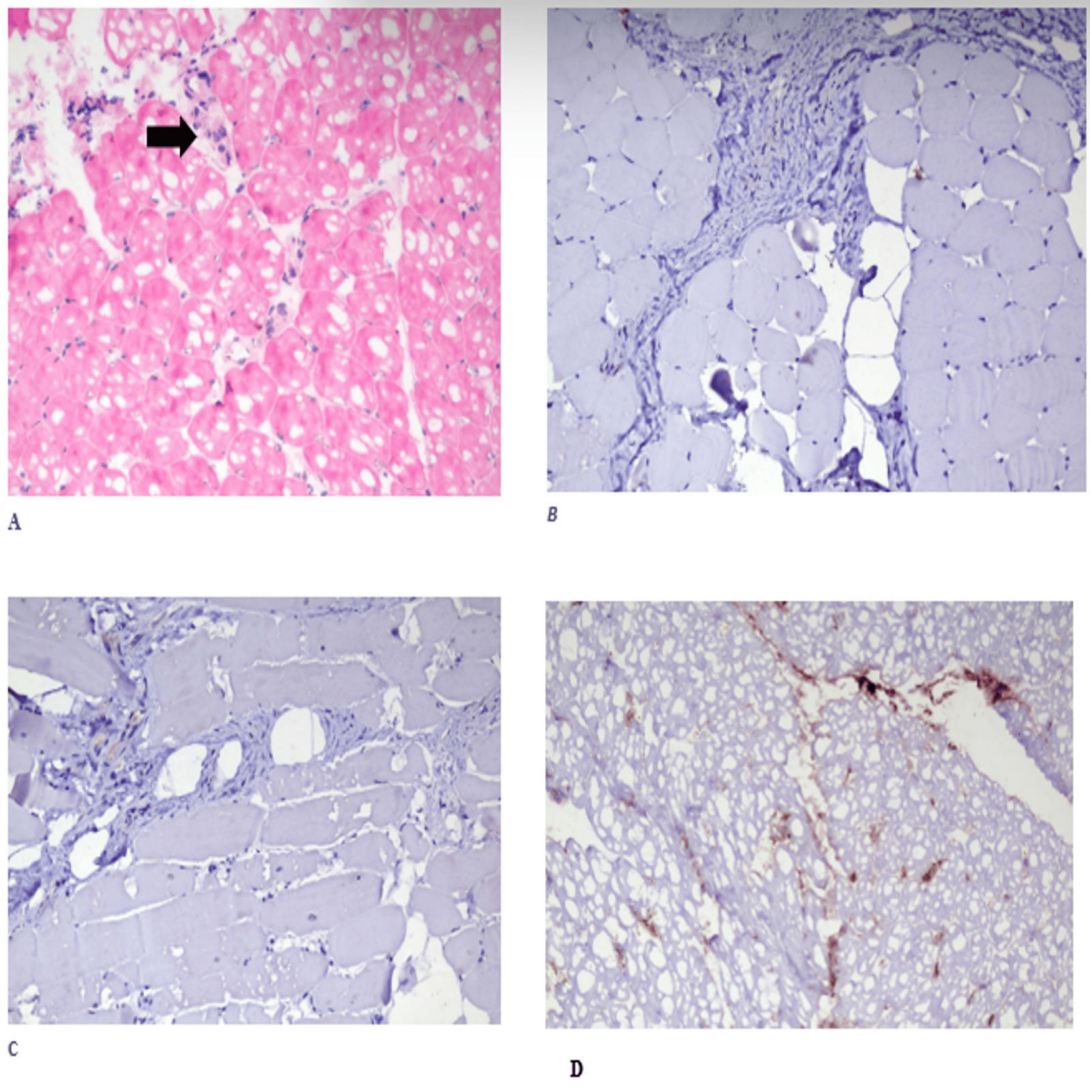
(A-D) Fragments of skeletal muscle tissue showing focal size variation in myofibers and mild lymphocytic infiltrates (×200; A: H&E, B: CD4, C: CD8, D: CD68) H&E, hematoxylin and eosin

Both eyes had 7 mm/5 min (normal range >5 mm/5 min) of moisture on Schirmer’s test. The nerve conduction study results were normal, but electromyography revealed myopathic patterns. Magnetic resonance imaging of the lower limbs showed diffusely elevated signal intensities in the quadratus femoris and anterior compartment muscles. A muscle biopsy of the rectus femoris was performed and stained using immunohistochemical stains with monoclonal antibodies (CD4, CD8, and CD68) (Figure 1). The biopsy showed myofibers of varying focal sizes and a few inflammatory cells, suggestive of inflammatory myopathy.

A diagnosis of myositis secondary to an autoimmune disorder (suspected SLE or Sjögren’s syndrome) was considered, although there was no definitive evidence to confirm either condition. HRCT of the chest was performed to assess for interstitial lung disease in support of a Sjögren’s syndrome diagnosis, but the results were normal. A 2D echocardiogram was also done and was normal. After reviewing the literature, we decided to initiate immunosuppressive therapy with prednisolone. The patient received intravenous prednisolone 60 mg once daily for three days, followed by oral prednisolone 60 mg once weekly for two weeks, with a plan for tapering. After one week of treatment, the patient showed significant improvement in muscle strength and normalization of muscle enzyme levels. She remained on regular follow-up with a tapering dose of prednisolone.

After 3 months of treatment, the ANA profile was repeated and showed ANA positivity (1:80, speckled pattern). Anti-dsDNA was also positive (92 IU/mL; normal: <30 IU/mL), with SSA/Ro60, SSB/La, and SSA/Ro52 all strongly positive (4+), while MPO, PR3, and anti-Scl-70 were negative, findings suggestive of active SLE. Her treatment was modified by adding HCQ and the steroid-sparing agent azathioprine. The patient was maintained on regular follow-up with prednisolone 2.5 mg once daily, HCQ 100 mg twice daily, and azathioprine 50 mg once daily. No new complaints or worsening of weakness were reported during follow-ups at one month, three months, one year, and two years.

## Discussion

SLE is a systemic autoimmune disorder characterized by diverse clinical manifestations, frequently complicating early diagnosis. Musculoskeletal involvement is prevalent, with arthralgia and non-erosive arthritis being the most commonly recognized symptoms [1]. Inflammatory myositis, while acknowledged within the range of lupus symptoms, is rare and seldom appears as the primary or only sign [3,4].

The patient demonstrated characteristic signs of inflammatory myopathy, including symmetrical proximal muscular weakness, increased muscle enzymes (CK and LDH), and myopathic EMG results. The lack of joint complaints, skin signs, or systemic characteristics originally rendered idiopathic PM the most probable diagnosis. Subsequent serological assessment demonstrated markedly elevated levels of antinuclear antibodies (ANA), anti-dsDNA, SSA/Ro 60, and SSB/La antibodies, thereby fulfilling the 2019 EULAR/ACR (European League Against Rheumatism/American College of Rheumatology) criteria for the diagnosis of SLE [7].

Distinguishing SLE-associated myositis from primary inflammatory myopathies is clinically crucial due to variations in prognosis and treatment. Although both illnesses may exhibit analogous muscular symptoms, SLE myositis is more prone to coexist with other systemic autoimmune characteristics and has a favorable response to corticosteroids [5,8]. Muscle biopsy in lupus myositis often reveals mild to moderate lymphocytic infiltration, muscle fiber destruction, and perivascular inflammation, but less pronounced than in dermatomyositis or PM [9].

Causes of initial anti-dsDNA negativity that subsequently transition to positivity include the delayed onset of autoantibody production as the disease progresses. Patients with idiopathic inflammatory myopathy may initially lack SLE-specific antibodies such as anti-dsDNA; however, these antibodies can emerge over time as the immune response evolves. Additionally, anti-dsDNA levels are known to fluctuate, often disappearing and reappearing in correlation with disease activity. This transient nature contributes to the low initial sensitivity of routine tests, which is approximately 52%-70% [10,11].

The treatment of SLE-associated myositis typically involves high-dose corticosteroids as the first-line intervention. In steroid-refractory cases, additional immunosuppressive agents such as methotrexate, azathioprine, or mycophenolate mofetil may be considered [12]. In our patient, the initiation of corticosteroids, hydroxychloroquine, and azathioprine led to significant improvement in muscle strength and normalization of muscle enzyme levels.

This case highlights the importance of including SLE in the differential diagnosis of inflammatory myopathies, particularly in young females. Prompt recognition and comprehensive autoimmune evaluation can facilitate early diagnosis and appropriate immunosuppressive treatment, thereby improving outcomes and preventing irreversible organ damage.

## Conclusions

This case exemplifies a rare and diagnostically challenging presentation of SLE, in which inflammatory myositis was the primary and sole clinical manifestation. Although musculoskeletal issues are prevalent in SLE, explicit myositis at the outset of the disease is rare and sometimes overlooked. Timely recognition of SLE-associated myositis is crucial, as it guides appropriate immunosuppressive treatment and helps prevent progression to multi-organ involvement and long-term damage. Clinicians must uphold a heightened suspicion for underlying connective tissue disorders in patients with idiopathic inflammatory myopathy, especially young females, despite the absence of typical lupus characteristics. Thorough autoimmune serology, corroborated by electromyographic and histological evidence, is essential for confirming the diagnosis. The prompt commencement of corticosteroids and disease-modifying medications in SLE-related myositis can provide positive results. This example underscores the need for a multidisciplinary approach and stresses the necessity for caution in assessing seemingly isolated symptoms that may indicate the emergence of a systemic autoimmune illness.
